# First Record of the Invasive Scale Insect, *Pulvinaria hydrangeae* Steinweden, 1946 (Hemiptera: Coccomorpha: Coccidae) in Romania

**DOI:** 10.3390/insects14040345

**Published:** 2023-03-31

**Authors:** Marius Paraschiv

**Affiliations:** National Institute for Research and Development in Forestry, “Marin Drăcea”, Cloșca Street 13, 500040 Brașov, Romania; marius.paraschiv@icas.ro

**Keywords:** *Pulvinaria hydrangeae*, cottony hydrangea scale, ovisac, invasive scale, pest species, *Acer* sp., *Tilia* sp.

## Abstract

**Simple Summary:**

Invasive insect species are organisms that cause significant damage to native flora and fauna after their introduction into a new geographic area, through human activity. Both the early detection and fast reporting of a new species in a country are essential. These enhance public knowledge and enable authorities to take measures to limit their spread and evaluate the potential risks that they pose. In this paper, we report on the apparition of a scale insect to Romanian fauna. This insect is native to Asia and has caused significant damage to the ornamental plants, shrubs and trees of Europe after its introduction to the continent. In Romania, this species was found on two native tree species: linden and sycamore. We believe that the species was brought to Romania via infested plants from abroad. In this paper, we summarize the worldwide distribution of this species and report on its hosts to highlight the pathways of infestation and to assess the potential risk that it poses to native flora. Moreover, we provide some morphological features of the species to aid in its identification in the field and in the laboratory.

**Abstract:**

Over the last few decades, globalization and global trade have increased the risk of the vehiculation of invasive organisms, which has had multiple negative effects, both economic and ecological. Through this study, we aimed to produce a report on the first record of the invasive scale insect *Pulvinaria hydrangeae* (Stein. 1946) in Brașov County in central Romania. It was found on two native tree species: sycamore (*Acer pseudoplatanus*) and linden (*Tilia cordata*). In this paper, we *(i)* highlight the list of possible hosts, *(ii)* provide a general outlook on infestations and *(iii)* review the control options for this particular pest. Because early detection and quick reporting are the most important actions in the successful management of invasive species, in general, we also provide a synthetic morphological description of the adult female specimens and ovisacs. Due to natural occurrence, our findings highlight the potential risks posed by the infestation of this insect to native tree species belonging to the Acer and Tilia genera. Because of the temperate climate in Romania and the fact that females are wingless, the new infestations will probably be made through the vehiculation of infested plants, rather than through natural spreading. However, because of global warming, the chances of this species surviving during the winter are likely to increase, making northern expansion of the cottony hydrangea scale feasible.

## 1. Introduction

Invasiveness represents one of the most important ecological threats to natural habitats, flora, fauna, and agricultural crops. This represents an action within an organism (plant or animal) that is vehiculated through human activity in other habitats, where they develop large populations and cause economic and ecological harm [1]. Globalization and increased global trade are considered to be the main causes for the spread of invasive organisms [2,3], rather than natural dispersion. Historically, European countries reported damages of EUR 116.61 billion between 1960 and 2020 [4]. However, in recent years across European Union countries, these organisms have been causing an economic loss of approximately EUR 12 million each year [5]. Moreover, on a global scale, the overall impact is huge: the damage caused by different insects is estimated to cost USD 70 billion per year [6]. One of the most problematic insect types are those that are herbivorous, and which produce multiple generations per season, enabling them to develop large populations in a short period of time. These could kill the hosts or cause them irremediable damage. Such species include those belonging to Coleoptera, Lepidoptera, Orthoptera, Hymenoptera and Hemiptera.

Scales insects are small, sap-sucking hemipteran parasites that live on plants across most of the world. They are important agricultural pests [7] and they differ from other insects through their protective waxy exudate—scale—after which they are named [8]. Phylogenetically, they are related to aphids, whiteflies and jumping plant lice, which all belong to the Sternorrhyncha suborder of the Hemiptera family [9,10].

Usually, the scales of these insects do not exceed 5 mm long, and they possess evident sexual dimorphism, in which the females are wingless and their reproduction is often parthenogenetic. Most of the species in this group reveal cryptic behaviors and exhibit invasive habits. Infestations of such species often cause damage to crops, trees and ornamental plants.

On a whole-world scale, the insect fauna comprises approximately 8200 types of the aforementioned species; however, this figure is generally believed to be larger [11]. In Europe, to date, there are known to be approximately 400–450 species of these insects, of which almost a third—129 species, belonging to 12 families—are considered to be alien to the continent [12]. Among these, Romanian scale fauna has been well-described, with 207 species currently recorded [13,14]. This is the fourth largest, after Italy (390 species [15]), France (381 species [16]) and Hungary (274 species [7]). Within this taxa, soft scales (Coccidae) are a special group, which are characterized by the waxy covering scales that are an integral part of their body. These differ from armored scales (Disipidae) and felt scales (Erioccocidae) through the fact that they excrete sugar honeydew, produce ovisacs and can move from branches to leaves [17], unlike those belonging to the other two genera. The Coccidae family is well represented, comprising 70 species. Of these, more than a third are considered invasive alien pest species, which are found on fruit trees and ornamental plants.

Among the newest invaders belonging to this family, the authors of [12] included the cottony hydrangea scale (CHS)—*Pulvinaria hydrangeae*—which is invasive to ornamental plants in urban areas. Some authors consider this insect to be a subtropical species, which is native to Asia [18,19]; however, others believe that North America is their native range [12]. Currently, this species is known to be present in 24 countries, across four continents: Asia, Europe, Australia and North America. It is a polyphagous insect and it is recorded as being present on 54 genera, belonging to 29 families of herbaceous plants, shrubs and trees [11].

Among the adult specimens, only the female is likely to be observed in nature because of their parthenogenetic reproduction, whereas the males’ role is relatively unknown, since they are alate but have no mouthparts [20].

The CHS has one generation per year all around Europe. During the winter they are immature, living as third instar nymphs in the canopies of trees, or at the apices of shrubs [21]. Some authors have suggested that the females die after laying their eggs [22]; however, it has been suggested that, in California’s climate conditions, they may spend the winter on the Hydrangea sp., specifically on the roots or the lower parts of the stems. The third instar is visible between October and April/May the following year; however, adults can be observed between April and June. The eggs, however, are only noticeable in June [23]. In most European habitats, the overwintering females become active in late spring and early summer. In this species, reproduction occurs through parthenogenesis [22,24]. The nymphs emerge from unfecundated eggs and then they feed on the underside of the leaves by sucking the sap. Furthermore, because the female nymphs resemble the adults, they are considered to be a paedomorphic species [9].

The nymphal development has three stages: The first two stages are spent under the leaves and occur during the entire summer. In the third stage, the nymphs leave the leaves just before they fall and climb on the twigs, where they aggregate and spend the cold season. Specific to this species, and also to the entire Pulvinarinii genera, is the production of a protective cover called an ovisac, which is used to protect the eggs. This takes the form of a white cottony “sac”, which is secreted by the female’s ventral tubular ducts, into which they lay the eggs. This ovisac sticks to the leaves and/or stems [21]. This could be several times the size of the female’s body length, as the females introduce their ovipositor inside it and start laying their eggs. Thus, the ovisac increases progressively through the female’s secretion [25].

Under large scale populations of these insects, some plant specimens can lack vigor and/or may shed their leaves. Furthermore, the aesthetic value of the plants can be affected, as a large number of ovisacs are visible on their leaves or branches [26]. In the literature, there are consistent reports of damage from across western Europe. This damage has been reported in plant nurseries, urban areas and natural habitats, and on a huge variety of hosts [27,28,29,30,31,32]. However, in other parts of the world, they are not causing such serious problems to the hosts. This is mainly because they are rare and their distribution is restricted to urban areas [25,33].

Because of this, we aimed to produce a report on the first recorded sighting of *P. hydrangeae* Steinweden, 1946, on Romanian fauna. The worldwide and European distribution of this insect, its invasive nature and the chronological pathways of infestations are also assessed. Moreover, in this paper, we *(i)* highlight a list of possible hosts in accordance with local flora, *(ii)* provide a general outlook on infestations and *(iii)* summarize the best control options for this pest.

## 2. Material and Methods

### 2.1. Study Area

**Figure 1:**
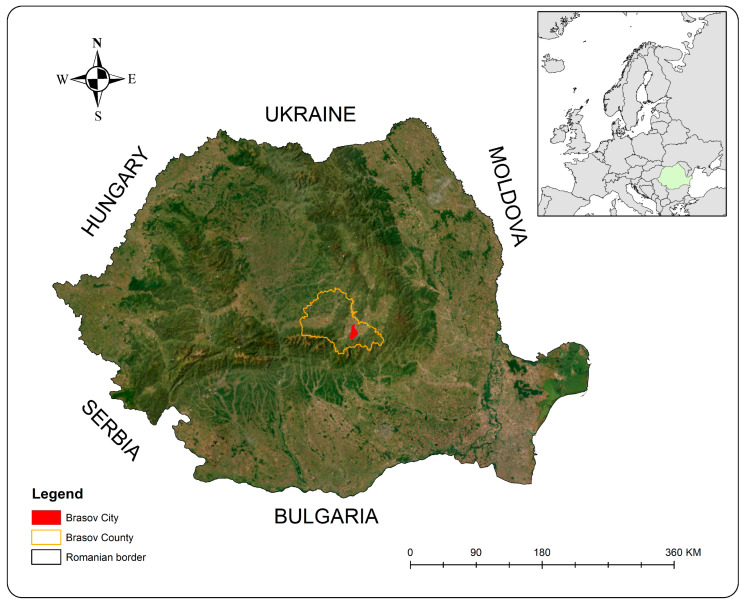
The study area (in red), Brasov County (in orange), Romania.

Geographically, Brașov County is located in the central region of Romania, southern Transylvania (Figure 1). Its orography is complex, comprising plains, hills and mountain massifs that frequently reach 2000 m altitude. The climate is influenced by atmospheric circulation from the north-west and is moderate-continental with an average annual temperature of 7–8 °C. Summer is approximately 50 days, with temperatures between 22 and 27 °C. Winter lasts longer, approximately 90 days, when temperatures can drop to approximately −18 (−20) °C. Average snow coverage lasts approximately 70 days; however, in recent years, snow has occurred mainly at the end of January and February. Annual rainfall precipitation is approximately 600–700 mm/year and atmospheric precipitation is approximately 75% [34,35].

The forests cover a significant part of the county’s area. According to altitudinal vegetation zones and forest typology, their management is mainly based on the natural regeneration of indigen species, exclusively. Among these, the most common tree species are sessile oak (*Quercus petraea* Matt.), beech (*Fagus sylvatica* L.), Norway spruce (*Picea abies* Karst.), fir (*Abies alba* Mill.), sycamore (*Acer pseudoplatanus* L.), hornbeam (*Carpinus betulus* L.), linden (*Tilia cordata* Mill.), etc. [35].

Brașov is the largest city in the county and is located in a depressionary area, at an altitude of 600 m a.s.l. The most important green areas are the natural forests bordering the southern part of the city. These are mostly administrated by the municipal forest service, which includes some forest reserves for scientific and recreational purposes or Sites of Community Interest (e.g., ROSCI0120 Muntele Tâmpa și Rezervația Naturală Tâmpa (Muntele) and ROSCI 0207 Postăvarul).

This is also an important industrial and trade center in Romania. It has large retail DIY chainswhich trade large amounts of imported ornamental plantsand significant wood processing factorieswhich use both local and imported conifer and broadleaf timber.

The urban green infrastructure of the city consists mainly of parks, squares, greenway trails, greenway street alignments, and public and private gardens. These are spread all over the town and are planted with many varieties of ornamental herbaceous plants, shrubs and trees.

### 2.2. Sample Collection and Laboratory Activities

Infested leaves (33 for each species) and twigs of sycamore (*A. pseudoplatanus*) and linden (*T. cordata*) were collected from Brașov’s urban area (N: 45°38′46.99″; E: 25°35′07.32″). The geographical coordinates (latitude and longitude) were recorded with a GPS handle—model Garmin 62 (Garmin Ltd., Olathe, KS, USA). In the laboratory, the females were gently removed from the ovisacs with a pin. Some of them were placed in Eppendorf vials (1.5 mL), containing 96% ethyl alcohol. The remainder were settled on watch glasses, at room temperature, for further examinations.

The remaining ovisacs on the leaves and twigs were visually inspected for general appearance, dimensions and parasites. Next, under a stereoscopic microscope (Stemi-508, Carl Zeiss, Oberkochen, Germany), the cottony ovisacs’ walls were opened with a pair of tweezers and an entomological pin to evaluate the eggs’ state. Eggs were checked for general aspect, color, relative size, chorion damage and presence of natural enemies (parasitoids). For each infested leaf (both hosts), an average dimensional ovisac was detached from the foliole and placed in 96% ethylic alcohol for 60 min. Next, several applications of α-pinene (99%) were made for 20 min to separate the eggs from the waxy secretion. Once this was dissolved, the remaining eggs were dried and counted under the microscope.

In total, eight females were mounted on Canada balsam, following the protocol in [25]. For identification, we used the morphological description and identification keys given in [26,36,37]. The specific features of the specimens were observed under the binocular microscope (Axio Imager A2—Carl Zeiss, Oberkochen, Germany). The dry specimens from the glass watch were investigated under the stereomicroscope to assess their length and width. The slides with mounted and dried specimens were deposited at the Forest Protection Laboratory, National Institute for Research and Development in Forestry, Brașov Station.

### 2.3. Data Analysis and Literature Survey

To investigate the differences between the infestation on both hosts (*A. pseudoplatanus* and *T. cordata*)—highlighted by the average number of ovisacs per leaf (two factors) and the average number of eggs per ovisac (two factors)—the variance was tested with ANOVA, with the level of significance established using Tukey test (Tukey’s multiple test). Before this, data were tested for normality and homogeneity with Student’s t-test. Statistical analyses were run under RStudio open-source software [38].

Distribution map was realized through ArcGIS version 10.6 [39], which integrated the worldwide records of the species from the literature with the GIS geodatabase. For some records (USA, Australia and China), it was possible to indicate the distribution in federal state or administrative regions; for the rest of them, this was conducted at the country level. Consequently, we overlayed those records onto the existent world countries map(inbuilt online ArcGIS accessibilities) and we exported the resulting distribution in “tiff” format (tagged image file).

For the literature overview, the available literature and web records were used to reconstruct the collection and hosts; although, for most of the records, only the year of the appearance was indicated.

## 3. Results

**Figure 2:**
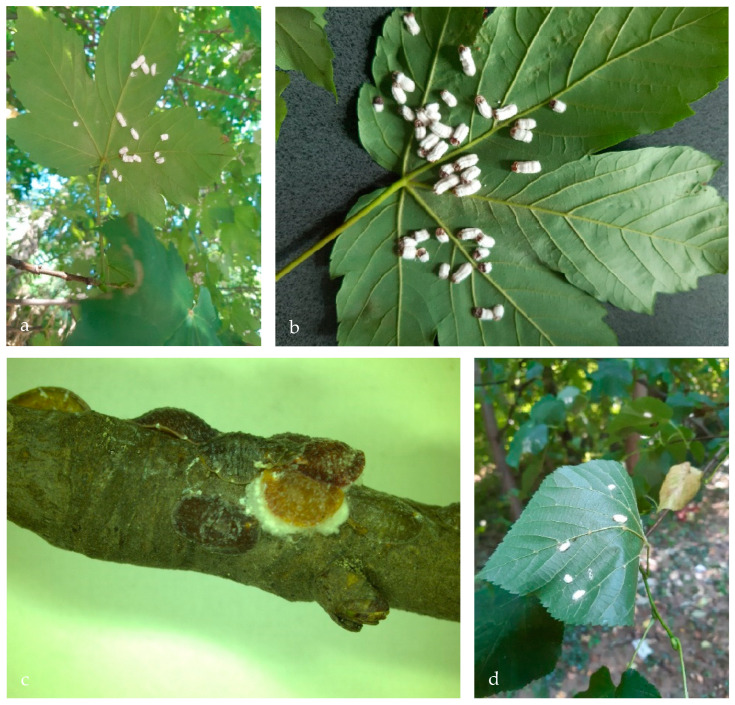
*P. hydrangeae* ovisacs on the underside of leaves of *A. pseudolpatanus* (**a**,**b**) and on the twigs and leaves of *T. cordata* (**c**,**d**).

For the first time in Romania, the CHS was recorded based on specimens collected from lindens (*T. cordata)* and sycamores (*A. pseudoplatanus)* (Figure 2). On both hosts species, the adult females and the laid ovisacs were recorded on the underside of the leaves. This was the only location of the ovisacs that we noticed on *A. pseudoplatanus*; however, on *T. cordata* we also noticed them on several twigs.

### 3.1. Material Examined

Location: Romania, Brașov City, Livada Poștei green area (N: 45°38′46.99″; E: 25°35′07.32″). From here, we obtained and examined the following: eight adult females with ovisacs on *A. pseudoplatanus* L., 20.05.2022, M. Paraschiv coll.; and six adult females with ovisacs on *T. cordata* Mill., 20.05.2022, M. Paraschiv coll.

### 3.2. Morphological Identification

**Figure 3:**
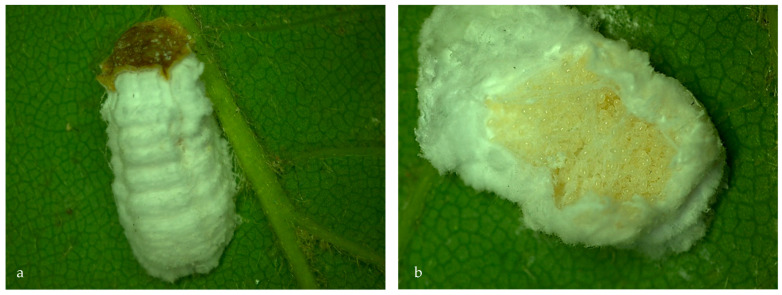
*P. hydrangeae* with ovisac: (**a**) visible parallel longitudinal strips and transversal ridges and (**b**) general aspect of the eggs laid inside the ovisac.

Living female: The females resemble an ovoid/a flat, circular shape. They are with 2–5 mm long and 1.5–2.0 mm in width. In the beginning, the females are yellow tobrown in color, or are mottled with brown; however, with age, they could become darker and develop transverse ridges [25,26,33,37,40]. The ovisacs are white, with a woolly texture. Convex, dorsally they are 10–15 mm long and 4–5 mm wide [41]. Longitudinally, the ovisacs are crossed by four ridges: two on each lateral side and two above them, parallel on the median areas (Figure 3). Moreover, small transversal ridges are visible perpendicular to the axis of the ovisac (Figure 3). These are believed to be made over time, as a consequence of the oviposition process inside them.

Slide-mounted females: Two accessible features can quickly identify the specimens that were mounted on the permanent medium, i.e., the presence of discal setae on the anal plates and the lack of dorsal tubercles.

Dorsum: The derm is of membranous consistency, with areolation evident but without tubercles. The setae are spinose—small and sharply pointed—and are positioned uniformly across the entire surface of the dorsum. Preopercular pores are positioned anterior to both anal plates and are clustered in elongate groups of more than 10. The anal plates are triangular in shape, with the long sides facing each other. There are four setae on each plate: one is in a subdiscal position and three are near the apex.

Margin: the marginal setae are curved, and the apices are acute, fimbriated or, rarely, bifid, with 10–20 setae on each side between stigmatic clefts.

Ventrum: The derm is of a membranous consistency, with visible multilocular disc pores (with 6–7 loculi) more abundant on the pregenital area but these decrease in abundance towards the median abdomen. These are also present toward the side of the meta- and mesocoxa. Three types of ventral tubular ducts are present: type I—broad ducts, with consistent ductule present, mainly on the medial area and submedially on the thorax and head; type II—in-between ducts, with slim interior ductule that are mainly spread on the abdomen, but which are also on the thorax; type III—small ducts that are positioned on the outward submarginal area.

In addition, the antennae are formed of eight segments and the legs are robust and have tibio-tarsal articulations.

### 3.3. Morphological Features

The difference between the average number of ovisacs on the leaves was significant (Df = 1, f = 2.530, *p* < 0.001): there were 16.2 per sycamore leaf but only 5.3 per linden leaf (Table 1). We did not find any statistical differences (Df = 3, f = 1.684, *p* = 0.380) between the female bodies that were collected from each host; the width of the bodies taken from each host was equal (1.7 ± 0.2), whereas length was 3.8 ± 0.2 for those collected from sycamore and 3.9 ± 0.3 for linden. The highest number of ovisacs was 53 per sycamore and 16 per linden. The ovisacs contained an average of 2136.8 eggs on *A. pseudoplatanus* and 1967.3 on *T. cordata,* with no statistical differences between them (Df = 1, f = 2.459, *p* = 0.264). However, the variation between the samples was noticeable: on the sycamore leaves, the minimum egg quantity was 1849 and the maximum was 2582; on the linden leaves, the minimum number of eggs was 1218 and the maximum was 1728. We did not find any parasitized eggs on the leaves from either host; however, we discovered an average of 6.3 eggs on the sycamore leaves and 8.6 on the linden leaves which had died from unknown causes. The general aspect of these eggs was of a darker color (almost brown–reddish), the chorion of the eggs was wrinkly and dehydrated, and the content was a liquid of a dirty white color.

### 3.4. Distribution and Historical Spreading

**Figure 4:**
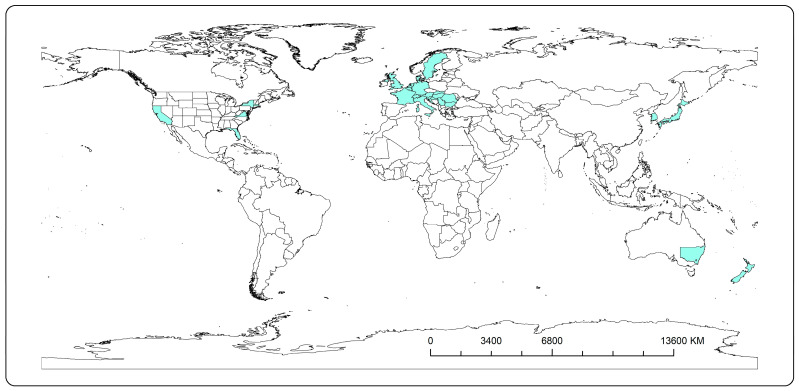
Updated distribution map of *P. hydrangeae* worldwide (green color); due to the size, the Canary Islands and Hong Kong are not visible here.

Including Romania, CHS is now present in 18 European countries (including the Canary Islands); five states in the USA (California, Florida, Massachusetts, New York and Virginia); the south of Australia (New South Wales); New Zealand; and Asia (Japan, Hong Kong (China) and South Korea) (Figure 4).

The data on the reports of the species can be divided into three periods.

The first phase coincides with the first description of the cottony hydrangea scale in Japan in 1907 and in the USA in 1946 (on samples collected in 1935 from Florida). At that time, the interest concerning this species was almost exclusively faunistic, as no significant damage reports were noted.

The second phase is represented by its introduction and spread across Europe. This was first noted in France in 1965, but it was 9 years until another report of it was made in Italy, in 1974. After another 9 years, in 1983, it was also reported in the Netherlands, and then, less than five years later, the species was also observed in the UK. This stage was characterized by the diversification of the hosts. It was during this stage that linden and sycamore were first mentioned as hosts (France, 1965, and Italy, 1976). In this interval, there were also some isolated reports of the CHS in Australia (1960) and New Zealand (1977). All of these records indicate that the infestations were of a disparate character, without any sign of the invasive nature of this pest (at least, not in these areas).

The third stage is represented by the period after the 1990s and up to the present day. During this interval, the species has been reported in 14 European countries, 9 of which reported its presence after the year 2000. This period is characterized by the mention of a varied spectrum of hosts. However, almost all of these records indicate at least one species of the genera Tilia and Acer, or both, as being hosts to the CHS. This crystallizes the theory that they have a preference for these plant species, at least on the European continent. Furthermore, in this interval the species has been reported in Hong Kong and South Korea (Table 2).

## 4. Discussion

The Pulvinaria species that have been recorded so far in Romania are *P. ampelopsidis* Săvescu, 1983; *P. brachiungualis* Săvescu, 1985; *P. corni* Savescu, 1985; *P. euonymicola* Săvescu, 1983; *P. floccifera* Westwood, 1870; *P. savescui* Săvescu, 1983; *P. regalis* Canard, 1968; *P. ribesiae* Signoret, 1873; *P. populi* Signoret, 1873; and *P. vitis* Linaeus, 1758 [13,56,74,75]. However, the latest synthesis [75] clearly validated the presence of only three species: *P. flocifera*, *P. regalis* and *P. vitis*. This is because the author considered the former records of *P. savescui* [76] (formerly *P. euonymicola*) as *P. regalis*. Moreover, *P*. *ampelopsidis*, *P. brachiungualis* and *P. corni* have not been recorded since their description by Săvescu in 1983 and 1985 [77,78]. This makes their identities uncertain and supports the suggestion made by the authors of [79] for an extended revision of the entire genus.

The nomenclatural history of the *P. hydrangeae* is complicated and has undergone recent changes. The first mention of the species was *P. kuwacola* [42], which was the name given to the specimen collected in 1985 from Japan. This was probably unknown in 1946, when the authors of [36] described the specimen collected from Florida, in 1935, which was named *P. hydrangeae.* This name was in use until 1960, when this was modified it into *P. fujisana* [43]; however, after a short time, the species was renamed into *Eupulvinaria hydrangeae* [41]. In 1993, the authors of [56] then revived the previously published combination: *P.hydrangeae*. Finally, in 2020, following the molecular investigation of the Coccidae family, the authors of [80] proposed that the valid name should be *P. kuwacola,* according to the first description given in [42].

In this study, our results in relation to the morphometric dimensions of the scales, eggs and ovisacs complied with the findings in [25,33], which indicated the length of the female’s bodies to be between 2 and 5 mm and the width to be 1.5–2 mm. There is no evidence that hosts or geographical regions affect the scale’s body dimensions. The fact that a higher number of ovisacs were observed on the sycamore leaves in our study is most probably because the area of these leaves is larger than those of the linden. Furthermore, the ovisacs’ dimensions and the female fertility was within the range indicated in the literature. However, the fact that we found differences in egg quantities between hosts is most probably due to the fact that laying was incomplete; the ovisacs were collected in May, and therefore the females had not finished the process of laying.

Because *P. regalis* (considered until 2018 as *P. savescui*) had not been recorded after its first mention in 1982, its presence in our country is doubtful. *P. hydrangeae* can be confused with *P. floccifera,* as suggested by the authors of [21]. Because both species have similar hosts and a similar biology, they could easily be confused. Therefore, the differentiation between them can only be conducted under a microscope, based on their morphological features. The authors of [25,50] considered the most evident features to be the presence of the subdiscal seta on each anal plate and the absence of dorsal submarginal tubercles in *P. hydrangeae*.

The fact that we discovered the adult female species in the spring of 2022 indicates that the cottony hydrangea scale was present in the investigated area at least one year before, in 2021. Our local investigation could not precisely determine the pathway of infestation. However, we are convinced that this was possible through infested ornamental plants from the surrounding areas, since the scale was discovered in the natural habitat, on indigenous trees.

Concerning the distribution and spread of this insect, in Europe we have noticed an expansion in the area of infestation towards the east of the continent. Moreover, over the last two decades, this trend has been very evident, as most of the countries where the species has been reported have been located in this geographical area. This is because, until the 1990s, these countries’ trade in plants and goods was generally quite restricted, for political reasons. However, with the exception of Serbia, where significant damage was recorded, in most of the other countries (e.g., Hungary, Croatia and Bulgaria) the presence, for the time being, is seen as faunistic. This fact contrasts strongly with the impact of the insect in the countries of Central and Western Europe (e.g., the Netherlands, Belgium and Luxembourg), where the species is causing significant damage. This suggests that there is currently a period of transition (acclimatization to the new conditions) for this species in Eastern Europe. On the other hand, its relatively recent discovery in South Korea and China (Hong Kong) could be an additional factor that suggests the Asian origin of this insect. Moreover, the facts that the main host, Hydrangeae sp., is native to Asia and the fact that, from 1935 until now, the species has only been observed in five states in the USA, supports this hypothesis.

To date, little is known about predators and parasitoids on this species, but some authors [27,55] indicate that two species of ladybugs (Coccinellidae) are predators for the CHS: *Adalia bipunctata* (L.) and *Exochomus quadripustulatus* (L.). *Coccophagus shillongensis* (Hayat and Singh) was observed by the authors of [81] as a parasitoid of the females and nymphs of *P.hydrangeae*. The encyrtid parasites, *Pseudococcus flavidus* (Kanda), were also found to be attacking this insect scale, by the authors of [82]. In natural habitats in Germany, the authors of [64] identified the chamaemyiid *Leucopomyia silesiaca* (Egg.) as egg predators, and the young larval stage and fungus *Verticillium lecanii* (Zimm.) pathogenic as predators of the female adults, larvae and eggs. Of these, we are only certain of the presence of both ladybugs in Romania’s fauna [83,84].

Laboratory tests carried out by the authors of [85] showed that the entomopathogenic nematodes *Steinernema carpocapsae* (Weiser), *S. feltiae* (Filipjev) and *Heterorhabditis bacteriophora* (Poinar) were virulent to the young CHS females; however, only *S. carpocapsae* was able to obtain mortality higher than 90%. Chemical control was investigated in [55]; however, the chemicals used by the researchers were mostly pyrethroids and organophosphorus pesticides, which are currently problematic for use in public urban areas. However, the authors of [21] suggested that, in order to be effective, the chemicals must be applied through spraying in midsummer, during nymphal development.

Due to its position in the middle of the country, its transport infrastructure and its importance as an industrial and trade center, Brașov city is very susceptible to the invasion of new pests. The records from the later years of [86,87,88,89], on both insects and pathogens, support this. Additionally, the natural occurrence of Aceraceae and Tiliaceae within the county could represent a risk factor—as was shown in the European records—because the CHS appears to prefer these species.

In the light of global warming and the growth of world trade, we expect this species to experience some outbreaks and also to be recorded in other places within Romania. It is probable that a warmer climate will increase the CHS’s chances of survival and favor its northern expansion, as indicated in [90]. However, as suggested in [91], in the near future this will be more restricted to human settlements because of the slightly increased temperature, compared to the surrounding areas.

## 5. Conclusions

In this study, we have produced the first reports of the presence of the CHS in Romania’s fauna, specifically, on two native host trees. This report increased the valid species belonging to Pulvinaria genus to four: *P. flocifera*, *P. hydrangeae*, *P. regalis and P. vitis*. However, our main conclusion was that the most susceptible hosts are those belonging to *Tiliaceae* and *Aceraceae*, due to the area’s climate specificity, the local conditions and the green areas’ infrastructure. Moreover, due to the increased trade in plants within the county, it is possible that the CHS will be reported on and may even cause outbreaks on other hosts in the near future. Therefore, for the moment, we are not able to evaluate the impact of the infestation; however, we consider that an assessment of this will be possible in the future, after the expansion of the CHS.

## Figures and Tables

**Table 1:** Morphometric features of *P. hydrangeae* female adults, ovisacs and eggs on host trees.

Host	Average Number of Females on Leaves	Females		Ovisacs		Average Number of Eggs/Ovisacs
		Length (mm)	Width (mm)	Length (mm)	Width (mm)	
*A. pseudoplatanus*	16.2	4.1	1.7	11.3	4.8	2136.8
*T. cordata*	5.3	3.9	1.7	11.1	4.7	1967.3

**Table 2:** Historical records of *P. hydrangeae* and the chronological infestation pathways.

Nr. crt	Country	Collection Data or Records *	Host	Source
1	Japan	**1907**, 1960	*Moraceae* sp., *Prunus indica, P. donarium and P. donarium* var. *spontanea.*	[42,43]
2	USA	**1946**	*Hydrangea hortensis.*	[36]
3	Australia	**1960**	-	[44,45]
4	France	**1965**	*Celtis australis, Cornus sanguinea, Diospyros kaki, Hydrangea macrophylla, Tilia platyphyllos, Tilia vulgaris, Morus alba, Platanus hybrida, Crataegus, Acer campestre, Acer monspessulanum, Acer pseudoplatanus.*	[46]
5	Italy	**1974**, 1987	*Acer negundo, A. platanoides, Chaenomeles japonica, Crataegus* sp. *Diospyros kaki, Deutzia* sp., *Hydrangea macrophylla, Lonicera caprifolium, Tilia platyphyllos.*	[47,48,49]
6	New Zealand	**1977**	*Hydrangea* sp., *Actinidia chinensis.*	[50]
7	Netherlands	**1983**, 1996, 2000	*Ilex* sp., *Viburnum opulus.*	[27,49,51]
8	United Kingdom	**1987**	*Hydrangea macrophylla, Malus* sp., *Prunus* spp., *Rheum* sp., *Lavatera* sp., *Acer* sp., *Viburnum* sp.	[52,53,54]
9	Belgium	**1990**, 1993	*Acer* sp. *Actinidia arguta, Aesculum* sp., *Amelanchier confuse, Aralia elata, Broussonelia papyrifera, Celtis* sp. *Cornus* sp., *Crataegus* sp., *Deutzia* sp., *Hydrangea hortensis, Magnolia* sp., *Malus* cf. *floribunda, Morus australis, Phellodendron* sp., *Prunus* sp., *Rosa* sp., *Rubus* sp., *Salix* sp., *Tilia* sp., *Viburnum* sp.	[55,56,57]
10	Slovenia	**1994**, 1998	*Acer palmatum, A. platanoides, A.pseudoplatanus, Cornus mas, C. sangunea, Hydrangea* spp., *Prunus avium, Taxus baccata, Tilia cordata, T.platthyphyllos, Actinidia chinensis.*	[29,58,59]
11	Switzerland	(1980?), **1994** (15.10.1994)	*Acer negundo*, *Taxus baccata, Hortensia* sp.	[60]
12	Canary Islands	**1995** (02.04.1995)	*Ficus* sp.	[61]
13	Germany	(1996?), **2000**, 2003	*Acer platanoides, Acer pseudoplatanus, Castanea sativa, Cornus mas, Ilex aquifolium, Tilia* sp., *Camelia* sp.	[62,63,64]
14	Luxemburg	**2000**	*Acer palmatum, A. platanoides, A. pseudoplatanus, Aesculus hippocastanum, A. pavia, Catalpa bignonioides, Carpinus betulus, Fagus sylvatica y compris* cv. *Pendula, Fraxinus excelsior, Magnolia* sp., *Platanus sp., Prunus* sp., *Quercus robur, Sorbus aria, S. aucuparia, S. intermedia, Tilia platyphyllos, T. tomentosa.*	[65]
15	Hungary	**2001**, 2013	-	[7,66]
16	Serbia	**2004**, 2011, 2013	*Hydrangea macrophylla and Tilia* sp.	[30,67]
17	Croatia	**2006**	*Acer campestre, Acer monspessulanum, Acer platanoides, Actinidia chinensis, Cornus mas, Photinia serrulta, Hydrangea macrophylla and Tilia cordata.*	[28,68]
18	Austria	**2007**	-	[69]
19	Bulgaria	**2010**	*Tillia* sp.	[70]
20	China	**2011**	*Celtis sinensis and Reevesia thyrosidea.*	[18]
21	Slovakia	**2014**	*Tlia* sp. *and Hydrangeae* sp.	[71]
22	Sweden	(2010?), **2014**	*Hydrangea* sp.	[72]
23	South Korea	**2015** (13.04.2015)	*Weigela* sp.	[37]
24	Poland	2016, **2018**	*Hydrangea macrophylla, Acer negundo, Acer platanoides, Cornus sanguinea and Tilia cordata.*	[26,73]

* bolded years represent the first mention; when the year is followed by (?), it means that its presence was observed, but without producing a report.

## Data Availability

Data are available upon request.
